# Antimicrobial Activity of a Phage Mixture and a Lactic Acid Bacterium against *Staphylococcus* *aureus* from Bovine Mastitis

**DOI:** 10.3390/vetsci7010031

**Published:** 2020-03-06

**Authors:** Isabel Titze, Volker Krömker

**Affiliations:** 1 Department of Bioprocess Engineering and Microbiology, Hannover University of Applied Sciences and Arts, D-30453 Hannover, Germany; 2 University of Copenhagen, Faculty of Health and Medical Sciences, Department of Veterinary and Animal Sciences, Section for Production, Nutrition and Health, Gronnegardsvej 2, DK-1870 Frederiksberg C, Denmark

**Keywords:** *Staphylococcus aureus*, bacteriophage mixture, phage therapy, lytic phage, lactic acid bacterium, dairy, bovine mastitis

## Abstract

The antimicrobial activity of a phage mixture and a lactic acid bacterium against *Staphylococcus aureus* isolates from bovine origin was investigated in vitro with regard to possible applications in the therapy of udder inflammation (mastitis) caused by bacterial infections. The *S. aureus* isolates used for inoculation derived from quarter foremilk samples of mastitis cases. For the examination of the antimicrobial activity, the reduction of the *S. aureus* germ density was determined [log_10_ cfu/mL]. The phage mixture consisted of the three obligatory lytic and *S. aureus*-specific phages STA1.ST29, EB1.ST11 and EB1.ST27 (1:1:1). The selected *Lactobacillus plantarum* strain with proven antimicrobial properties and the phage mixture were tested against *S. aureus* in milk, both alone and in combination. The application of the lactic acid bacterium showed only a low reduction ability for a 24 h incubation period. The bacteriophage mixture as well as its combination with the lactic acid bacterium showed high antimicrobial activity against *S. aureus* for a 24 h incubation period at 37 °C, with only the phage mixture showing significance.

## 1. Introduction

The therapy of intramammary infections (IMIs) in conventional dairy farming involves the use of antimicrobial agents for treatment during lactation and for selective drying off [1,2]. A targeted use of antibiotics is necessary to ensure the health and well-being of animals and to achieve a high level of food safety. Nevertheless, bacterial resistance against antimicrobial agents in human as well as veterinary medicine and increasing public concern about the usage of antibiotics in food-producing animals make it necessary to further reduce their application [3,4,5].

*Staphylococcus (S.) aureus*, a Gram-positive, catalase-positive and coagulase-positive bacterium, represents one of the most important mastitis pathogens [6,7]. Although environment-associated pathogens have become increasingly important, there are still significant aspects to consider, such as the remediation of *S. aureus* at the herd level, which still represents a long-term and labor-intensive process. As a cow-associated major pathogen, *S. aureus* is spread between animals, especially during the milking process [7,8,9]. Moreover, characteristics such as low cure rates and a long persistence in the mammary gland make it a difficult-to-treat udder pathogen whose sanitation requires additional hygiene and management efforts [4,7,10]. Control programs covering aspects of milking hygiene, separation of infected animals and therapeutic measures are therefore particularly important [11,12]. Dufour et al. used multilevel regression models to investigate the risk factors associated with prevalence, incidence during lactation and elimination of *S. aureus*-induced bovine IMI [13]. Key factors related to low prevalence in the herd include a low incidence as the most important factor on herd level and a high elimination rate of *S. aureus*. The incidence of *S. aureus* IMI was mainly associated with milking procedures (hygiene measures: wearing gloves, disinfection of teats before milking; good condition of teat ends). These factors are simultaneously associated with the reduction of the prevalence and the elimination of *S. aureus* [13].

The pathogenicity factors enable *S. aureus* to perform encapsulation, intracellular occurrence, adhesion to epithelial cells and biofilm formation [14]. This often leads to subclinical or chronic inflammation of the bovine mammary gland, impedes the effect of an antibiotic treatment and might be the cause of insufficient cure rates for an antibiotic treatment during lactation [7,15]. Therefore, antimicrobial alternatives are of particular interest to increase the bacteriological cure (BC) rates during lactation while significantly reducing the application of antibiotics [16].

The use of bacteriophages (phages) for the treatment of bacterial infections (bacteriophage therapy) represents such an alternative approach. Several studies in animal models have already investigated bacteriophages, with promising results regarding a therapeutic use [17,18,19,20]. Phages hold various advantages over conventional antibiotic agents, as they are very host specific and thus protect the physiological bacterial flora [21,22]. Furthermore, new phages can be isolated within a comparatively short time. This advantage, the possibility of combining different phages in the form of a bacteriophage mixture, as well as the possibility to combine phages and antibiotics reduce the risk of a resistance development against single phages [23,24,25]. Various studies indicate a prolonged antibiotic therapy of *S. aureus*-induced intramammary infections (IMIs) to improve bacteriological cure rates, resulting in an increased use of antimicrobial agents [26,27,28]. For this reason, alternative treatment methods such as phage therapy could be particularly useful to reduce the amount of antibiotics administered.

Lactic acid bacteria (LAB) are a heterogeneous group of Gram-positive, catalase-negative and facultative anaerobic microorganisms which are summarized due to their specific kind of metabolism [29,30]. LAB also possess antimicrobial properties resulting from the colonization of epithelia, the competition for nutrients and the production of organic acids such as lactic acid, free fatty acids as well as hydrogen peroxide, diacetyl, bacteriocins or bacteriocin-like substances [31,32,33]. Furthermore, LAB are able to modulate the host’s immune system [34]. The term “probiotic”, which is closely associated with LAB and bifidobacteria, is used in the sense of “health promoting” and “probiotics” are, by definition, living microorganisms with a consumer health benefit when properly administered [29,31,35]. Several in vitro and in vivo studies on the antimicrobial activity of LAB against *S. aureus* were published [36,37,38]. A number of studies on the use of LAB for the therapy of bovine udder infections have already been carried out, with promising results [39,40,41]. Crispie et al. (2004) even described the use of a lacticin produced by the probiotic *Lactococcus lactis* DPC 3147 as effective as a conventional antibiotic treatment for dry cow therapy [42]. 

*Lactobacillus plantarum* is a facultative heterofermentative LAB, which is mostly isolated from dairy products, sewage and from human or clinical origin [30,43]. *Lb. plantarum* strains are known for their probiotic properties, including the formation of a specific bacteriocin, called plantaricin [37,44,45,46].

The aim of the present study was the in vitro investigation of the antimicrobial activity for the combination of an obligately lytic bacteriophage mixture (PM) and the *Lb. plantarum* strain 118/37 against *S. aureus* isolates from mastitis cases. The *Lb. plantarum* strain 118/37 has been extensively tested in previous examinations and has proven its antimicrobial activity against *S. aureus* [47]. The examinations were conducted regarding a combined application in vivo for the therapy of *S. aureus* mastitis during lactation.

## 2. Materials and Methods 

### 2.1. S. aureus Strains and Growth Conditions

The *S. aureus* isolates 7142 and 10614 used for inoculation of ultra-high temperature (UHT) milk were isolated from two different dairy farms in Germany and provided by the Department of Bioprocess Engineering and Microbiology, University of Applied Sciences and Arts Hannover (Germany). They were obtained from quarter foremilk samples of mastitis cases and were tested as coagulase-positive as well as screened *nuc* gene-positive. To confirm these results for the present study, species affiliation was performed using matrix-assisted laser desorption time-of-flight-mass spectrometry (MALDI-TOF), resulting in *S. aureus* for both isolates. The isolates were stored at −80 °C with addition of 20 % glycerin after a 24 h culture in brain heart broth and plated on esculin blood agar (Oxoid Deutschland GmbH, Wesel, Germany) (24 h at 37 °C) before use. 

The *S. aureus* strains ST11, ST27 and ST29, isolated and provided by the Phage Technology Center (PTC) GmbH, Bönen (Germany), were used for routine phage propagation (Table 1). The strains ST11 and ST27 originated from mastitis cases. The phage propagation strains were stored at −80 °C with addition of 20 % glycerin and plated on esculin blood agar (Oxoid Deutschland GmbH, Wesel, Germany) before use. Luria–Bertani (LB) broth (trypton/pepton form casein: 1 g/100 mL; yeast extract, micro-granulated: 0.5 g/100 mL; sodium chloride (NaCl): 0.5 g/100 mL; Carl Roth GmbH & Co. KG, Karlsruhe, Germany) was used for bacterial growth in suspension and for phage plaque assay. LB broth was supplemented with agar (Agar Agar *Bioscience*; Carl Roth GmbH & Co. KG, Karlsruhe, Germany) at concentrations of 1.5 % for bottom and 0.5 % for top agar and sterile dextrose was added at 1.1 % to the top agar before use. Baird–Parker agar (Carl Roth GmbH & Co. KG, Karlsruhe, Germany) supplemented with egg yolk tellurite-emulsion (Carl Roth GmbH & Co. KG, Karlsruhe, Germany) was used for differential counting of *S. aureus* germ density. 

### 2.2. Lactic Acid Bacterium and Growth Conditions

The lactic acid bacterium (LAB) 118/37 identified as *Lactobacillus* (*Lb.*) *plantarum* is an isolate from bovine milk and was provided by the Department of Bioprocess Engineering and Microbiology of the University of Applied Sciences and Arts Hannover (Germany). It has been isolated and described in a research project, tested for its antimicrobial properties and patented. Details can be found at Diepers et al. (2017) [47]. The LAB was stored at −80 °C with addition of 20 % glycerin after a 24 h culture in de Man, Rogosa and Sharpe (MRS) broth (Merck, Darmstadt, Germany). It was cultured twice in MRS broth (37 °C, 24 h, anaerobically) and simultaneously plated on MRS-agar (Merck, Darmstadt, Germany) for optical detection of purity before every use. Species identification based on microbiological identification for Gram-positive, catalase-negative, oxidase-negative (*Bactident* Oxidase; Merck, Darmstadt, Deutschland), fermentative (OF basal medium with addition of D(+)-glucose monohydrate; Merck, Darmstadt, Deutschland), H_2_S-negative, immotile rods. Further biochemical and genetic species identification was performed by Diepers et al. (2017) and based on analytic profile index (API) assay and the detection of 16SrDNA sequence according to Kwon et al. (2004) [47,48]. To confirm these results for the present study, species affiliation was performed additionally using MALDI-TOF, resulting in *Lb. plantarum* for the LAB strain 118/37. 

LAB 118/37 was chosen for the present examinations according to safety requirements (no resistance coding genes) and properties such as the proven antimicrobial activity against *S. aureus* ATCC 12600 and the adhesion to teat canal epithelial cells in vitro [47]. 

### 2.3. Bacteriophages

Bacteriophages STA1.ST29, EB1.ST11 and EB1.ST27 were isolated and provided by PTC GmbH, Bönen (Germany). These lytic, sequenced phages belong to the order of *Caudovirales* and the families of *Myoviridae* (STA1) and *Podoviridae* (EB1) (Table 1). Phage STA1 is an isolate of a wastewater facility and is closely related to Phage K, which was isolated and characterized by O’Flaherty in 2004 [49]. Phage EB1 is an isolate from pig manure and is closely related to the phage PSa3, which was isolated and characterized by Kraushaar et al. in 2013 [50]. The genetically distinct phages were grown on different host strains (ST29; ST11 or ST27), which might have led to epigenetic modifications [51] that resulted in a different host range in previous examinations [52].

A bacteriophage mixture (PM) was prepared to enlarge the host range for the examination in UHT milk and with regard to an in vivo use and to decrease the risk of a resistance development against single phages [25]. It contained phages STA1.ST29, EB1.ST11 and EB1.ST27 in equal quantities and a final concentration of 7.4 × 10^9^ pfu/mL. The phages were selected according to previously examined criteria including a broad host range against *S. aureus* isolates from mastitis cases, good propagation properties and adequate storage stabilities [52]. All phages of the mixture showed lytic activity against the *S. aureus* isolates 7142 and 10614 in the plaque assay and the phage mixture (PM) showed antimicrobial activity against the *S. aureus* isolates in pasteurized and raw milk in previous examinations [52]. 

### 2.4. Bacteriophage Propagation

For phage propagation, LB broth was inoculated with 1 % of an overnight culture of the respective *S. aureus* strain (ST11, ST27 or ST29). The optical density at 600 nm (OD_600_) was determined by a photometer (SPEKOL^®^1500, Analytik Jena AG, Jena, Germany) every 30 min and one percent of a phage stock solution with a concentration ≥ 1.5 × 10^10^ pfu/mL (EB1.ST11), ≥ 1.3 × 10^9^ pfu/mL (EB1.ST27), ≥ 1.6 × 10^9^ pfu/mL (STA1.ST29) was added when an OD_600_ of 0.3 was reached. The OD_600_ was measured at half-hourly intervals until a decrease could be seen. Chloroform was added at 1 ‰ and the solution was centrifuged (10,000× *g* for 20 min at room temperature) before supernatant was removed and sterile filtered (using a filter with a pore size of 0.45 µm, Minisart^®^NML Plus/NY Plus, Sartorius, Göttingen, Germany). The plaque forming units per milliliter (pfu/mL) were determined by using a double-layer agar technique modified to the one described by Sambrook and Russel (Sambrook and Russel 2001). Briefly, 100 µL of the phage serial dilution (Ringer’s solution; Merck KGaA, Darmstadt, Germany) was added to 100 µL overnight culture of the respective *S. aureus* propagation strain and incubated for 5 min at room temperature. After addition of 5 mL top agar, the assays were mixed by a Vortex Mixer (Vortex Genie^®^2; Scientific Industries, Bohemia, NY, USA) and poured onto a petri dish (Ø94 × 16 mm) prepared with 10 mL LB—bottom agar. The inverted plates were incubated for 18 h at 37 °C until the plaque forming units per milliliter (pfu/mL) were determined. The phage solution was stored in the dark at + 6 °C until further investigation. 

### 2.5. Antimicrobial Activity of a Phage Mixture and a Lactic Acid Bacterium in Milk

With regard to a future use in the therapy of *S. aureus* mastitis in dairy cows, we investigated the antimicrobial activity of a phage mixture (PM) and a lactic acid bacterium in milk for an approximation to in vivo conditions. The antimicrobial activity was examined for a single in comparison to a combined use of the PM (STA1.ST29, EB1.ST11 and EB1.ST27) and the *Lb. plantarum* strain 118/37 (LAB) against two *S. aureus* isolates from mastitis cases, respectively, and was determined by the reduction of the *S. aureus* germ density [cfu/mL]. Baird–Parker agar plates were used to prevent contamination by the lactic acid bacterium, representing a selective culture medium for the isolation of coagulase-positive *Staphylococcus* species. The *S. aureus* isolates 7142 and 10614 were tested for their typical growth (black colonies surrounded by a clear zone) on Baird–Parker agar before trial. Commercial ultra-high temperature (UHT) milk was used to achieve high reproducibility and to prevent contamination by the natural milk microflora. The milk was tested for absence of *S. aureus* and antibiotic residues by direct plating and a Brilliant Black Reduction Test (Delvotest^®^BR Brilliant, DSM; MILKU Tierhygiene GmbH, Bovenden, Germany) prior to examination. 

The UHT milk was inoculated with *S. aureus* isolates 7142 or 10614 respectively at an average concentration of 1.3 × 10^5^ cfu/mL. Depending on the approach a) the PM at a concentration of 1.3 × 10^9^ pfu/mL, b) the LAB (*Lb. plantarum* 118/37) at a concentration of 6.6 × 10^6^ cfu/mL or c) a combination of PM and LAB was added, resulting in a total amount of 7 mL for each approach. Previous examinations [52] have shown the best reduction ability for the concentrations of *S. aureus* isolates (7142; 10614) and PM, which were also used in the present trials. For the application of *S. aureus* and LAB, a ratio of 1:10 was used according to the findings of previous studies [53,54].

The pH value was constantly neutralized to prevent germ reduction solely due to acid formation by the LAB and in order to investigate the effect of other products such as antimicrobial peptides produced by the LAB. NaOH (1 mol/L) was used to achieve a constant pH value between 6.0 and 7.5. A fourth approach was solely used to determine the pH values and the amount of NaOH to be added.

Samples were taken after 30 min, after 12 h and after 24 h of incubation at 37 °C under aerobic conditions. Serial dilutions (RINGER, Merck, Darmstadt, Germany) were examined for their *S. aureus* germ density by using the spatula method with 100 µL sample material on Baird–Parker agar plates with addition of egg yolk tellurite (Baird-Parker-Agar, Basis, ROTH; egg yolk tellurite, ROTH) according to DIN EN ISO 6888-1. The 24 h incubation period was chosen according to common growth characteristics of LAB in milk and to investigate a possible long-term effect of the LAB on *S. aureus* [55,56]. To obtain statistically relevant data, the trial was carried out in triplicate including controls without phages and LAB (solely *S. aureus* isolate 7142 or 10,614) and negative controls (UHT milk without additives; solely LAB). 

### 2.6. Statistical Analysis

Three measurements were collected for each data point. A statistical calculation was performed to determine the influence of the PM, the LAB and their combination on the reduction of the *S. aureus* germ density in UHT milk. The values of the *S. aureus* germ density (cfu/mL) were log transformed (log_10_) and one was added to approximate normal distribution. The data were analyzed by ANOVA using SPSS 25.0 (IBM SPSS 25.0.0.0., Armonk, NY, USA). *p* values < 0.05 were considered significant.

## 3. Results

### Antimicrobial Activity of a Phage Mixture and a Lactic Acid Bacterium in Milk 

**Figure 1:**
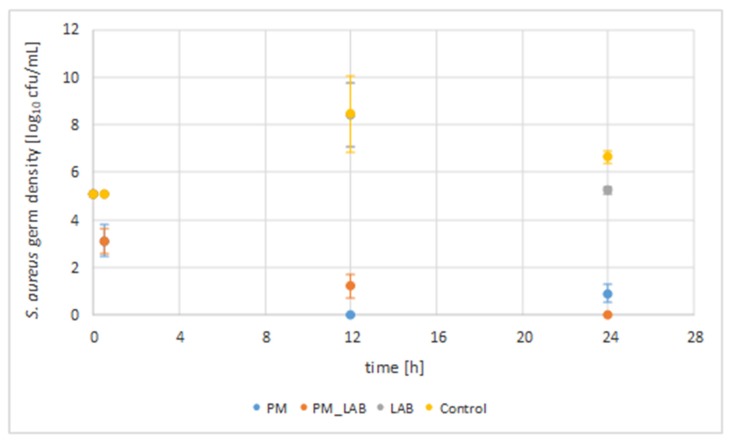
*S. aureus* germ density for the application of a phage mixture, a lactic acid bacterium and their combination. The *S. aureus* germ density [log_10_ cfu/mL] for 100 µL sample material is given with standard errors. The common mean values of the two *S. aureus* isolates 7142 and 10614 are given (SEM 5.25 log cfu/mL ± 0.15 SEM) as the statistical calculation showed no significant difference between the two *S. aureus* isolates (*p* > 0.05). * Time of incubation at 37 °C in ultra-high temperature (UHT) milk. PM = phage mixture (STA1.ST29; EB1.ST11; EB1.ST27) (*p* < 0.05); LAB = lactic acid bacterium (118/37, *Lb. plantarum*) (*p* > 0.05); PM + LAB = combined application of the phage mixture and the lactic acid bacterium (*p* > 0.05).

The combination of a phage mixture and a lactic acid bacterium as well as their solitary use were investigated in UHT milk for their antimicrobial activity against *S. aureus* isolates 7142 and 10614 (initial concentration of 1.3 × 10^5^ cfu/mL), respectively (Figure 1). Samples were taken after 30 min, 12 h and after 24 h. The germ density was calculated as cfu/mL and is given as a logarithm of the common mean values of the two *S. aureus* isolates [log_10_ cfu/mL] used for inoculation in the different approaches, as the statistical calculation showed no significant difference between the two isolates (*p* > 0.05).

The control without phages and LAB showed an average increase of 3.3 log units in *S. aureus* germ density for incubation at 37 °C to an average value of 8.5 log cfu/mL (SEM 5.25 log cfu/mL ± 0.15 SEM) after 12 h. After 24 h of incubation, an average germ density of 6.7 log cfu/mL (SEM 5.25 log cfu/mL ± 0.15 SEM) of *S. aureus* was observed. 

The sole addition of PM resulted in a reduction (*p* < 0.05) compared to the initial germ density (5.1 log cfu/mL) by an average of 1.9 log units reduction to a value of 3.2 log cfu/mL (SEM 5.25 log cfu/mL ± 0.15 SEM) after 30 min, a reduction by 5.1 log units to a value of 0.0 log cfu/mL (SEM 5.25 log cfu/mL ± 0.15 SEM) after 12 h and a reduction by averagely 4.2 log units to a value of 0.9 log cfu/mL (SEM 5.25 log cfu/mL ± 0.15 SEM) after 24 h of incubation at 37 °C (Table 2). Compared to the control without phages and LAB at the same time, the reduction of the *S. aureus* germ density (*p* < 0.05) was 1.9 log units (30 min), 8.5 log units (12 h) and 5.8 log units (24 h).

The addition of the LAB alone did not lead to a reduction of the microbial density compared to the initial value for a 24 h incubation period, but to an increase by 3.3 log units (12 h) to a value of 8.4 log cfu/mL (SEM 5.25 log cfu/mL ± 0.15 SEM) and an increase by 0.1 log units (24 h) to a value of 5.2 log cfu/mL (SEM 5.25 log cfu/mL ± 0.15 SEM). Nevertheless, a reduction by <1 log unit after 12 h and by averagely 1.5 log units was shown compared to the control without phages and LAB after 24 h (*p* > 0.05). 

The combined application of PM and LAB resulted in a reduction of the initial germ density (*p* > 0.05) by an average of 2.0 log units (30 min) to a value of 3.1 log cfu/mL (SEM 5.25 log cfu/mL ± 0.15 SEM), by 3.9 log units (12 h) to a value of 1.2 log cfu/mL (SEM 5.25 log cfu/mL ± 0.15 SEM) and by 5.1 log units (24 h) to a value of 0.0 log cfu/mL (SEM 5.25 log cfu/mL ± 0.15 SEM). Compared to the control without phages and LAB at the same time, the combined application led to a reduction (*p* > 0.05) by 2.0 log units (30 min), 7.3 log units (12 h) and 6.7 log units (24 h).

In conclusion, it can be said that the application of PM led to a considerable reduction of the bacterial density over a period of 24 h in contrast to the sole application of LAB. The statistical calculation showed significance for the use of PM (*p* < 0.05), but no significance for the application of the LAB or the combination of both (*p* > 0.05). Thus, it can be concluded that the addition of LAB had no additional effect on the reduction of the *S. aureus* germ density for an incubation period of 24 h.

## 4. Discussion

*S. aureus* strains possess certain pathogenicity factors that hamper antibiotic treatment and lead to insufficient bacteriological cure rates during lactation [7,15]. Research into alternatives for the treatment of *S. aureus* mastitis is therefore an important component in improving cure rates, reducing antibiotic consumption and thus reducing the risk of developing bacterial resistance. Lytic phages and probiotic lactic acid bacteria are regarded as alternative approaches in the therapy of bacterial infections.

For this reason, we investigated the combination of a three-component lytic bacteriophage mixture of sequenced *Myo*- and *Podoviridae* and a wild-type *Lb. plantarum* strain against *S. aureus* isolates from mastitis cases. Since obligatory lytic phages usually lead to a rapid reduction of pathogens, while lactic acid bacteria proliferate more slowly, but can establish themselves in the target tissue for longer, this combination was assumed to have an advantage for the treatment of *S. aureus* mastitis compared to use alone. Thus, an incubation period of 24 h was chosen considering the slower growth and the delayed production of antimicrobial peptides by the LAB. The present in vitro examinations were conducted with regard to a combined use in mastitis therapy. The *S. aureus* isolates used for inoculation were already investigated in a previous study and the PM also used in the present study was able to significantly reduce the *S. aureus* isolates in pasteurized and raw milk [52].

Bacteriophages are viruses that exclusively target prokaryotic cells. Obligately lytic phages in particular are able to proliferate inside and lyse the host cell within a short time. Phages commonly show very host-specific targeting, which leads to protection of the apathogenic flora in the target tissue when administered in vivo [21]. Several examinations in animal models have already demonstrated that the use of bacteriophages in vivo leads to a significant pathogen reduction without adverse effects on the treated animals [17,18,20].

Similar to any antibacterial agent, bacterial resistance can develop against phages. However, the possibility for a comparatively rapid isolation of new phages, the use of obligately lytic and sequenced phages with a lack of lysogenic potential, the usage of several phages in a mixture and finally the opportunity for a combined use of phages and antimicrobial agents reduce the risk for resistance development and represent advantages over common antibiotics [23,25,57,58]. In the present study, we used a mixture of obligately lytic, sequenced phages to fulfil functional and safety requirements. The phages of the mixture were already investigated in a previous study, resulting in a broad host spectrum against *S. aureus* isolates from mastitis cases [52]. 

Lactic acid bacteria also possess the ability to inhibit the growth of pathogens by the colonization of epithelia, the competition for nutrients and the production of antimicrobial active substances [30,31,32]. *Lb. plantarum* strains are known for their probiotic properties partly due to their production of a bacteriocin called plantaricin [44,45,46]. The literature describes general properties of lactic acid bacteria intended to be used as probiotics. These include a proven antagonistic activity against pathogens, the adhesion to and the persistence on epithelia, as well as the survival and a maintained functionality in the target tissue [34]. In addition, the modulation of the host’s immune response represents an important probiotic property [34,59,60]. Safety aspects include the absence of resistance-coding genes and a general susceptibility to antibiotics in case of treating immunosuppressed patients [34]. The LAB 118/37 used in the present study was isolated from milk and was already examined by Diepers et al. (2017) [47]. This *Lb. plantarum* strain was selected for the present examinations based on its properties, such as a proven antimicrobial activity against *S. aureus*, the possibility of using milk as a nutritional substrate and the ability to adhere to mammary gland canal epithelial cells in vitro [47]. In addition, its lack of resistance-coding genes fulfills an important safety requirement [34,47].

In the present study, we investigated the antimicrobial activity of a single and a combined use of the PM and the LAB against *S. aureus* isolates in UHT milk. The *S. aureus* isolates originated from quarter foremilk samples of mastitis cases and thus present typical mastitis causing pathogens. The antimicrobial activity was evaluated for the reduction of the *S. aureus* germ density in relation to the initial germ density (t_0h_), and in relation to the values of the control culture without phages after the same incubation period. The statistical calculation showed significance only for the use of PM. The evaluation of the results has already shown an effect on the germ density at the beginning of the experiment (t_30min_). Both the PM (*p* < 0.05) and the combination of PM and LAB (*p* > 0.05) led to a reduction of the *S. aureus* germ density in UHT milk after 30 min compared to the initial value, which increased to an average reduction by 5.1 log units (PM) (*p* < 0.05) and 3.9 log units (PM + LAB) (*p* > 0.05) after 12 h. Compared to the initial values, the reduction ability decreased slightly after 24 h when using PM alone (reduction by 4.2 log units; *p* < 0.05), but increased further when combining PM and LAB after 24 h incubation (reduction by 5.1 log units; *p* > 0.05). The highest reduction compared to the initial values was thus shown after 12 h for the use of PM (*p* < 0.05) and after 24 h for the combination of PM and LAB (*p* > 0.05).

A constant reduction was also shown in comparison to the controls without phages, with the highest reduction by 8.5 log units for PM (*p* < 0.05) and by 7.3 log units for the combination (*p* > 0.05) after 12 h. The use of the LAB alone led to no reduction when compared to the initial values for an incubation period of 24 h, but to a slight increase in germ density. Nevertheless, compared to the control culture without phages, the LAB led to a reduction of the *S. aureus* germ density by 1.5 log units after 24 h (*p* > 0.05). 

It can therefore be said that for an incubation period of 24 h, only the use of PM led to a significant reduction in germ density (*p* < 0.05). The combination of PM and LAB was more successful than the use of LAB alone in terms of antimicrobial activity against *S. aureus* in UHT milk, but both did not show significance (*p* > 0.05). 

The results of the present investigations are partly comparable to the results of Woo and Ahn (2014) for their examination of a combined use of phage SA11 and a *Lactobacillus (Lb.) rhamnosus* strain against *Staphylococcus aureus* for modeled intestinal conditions in vitro at different pH values [61]. Phage SA11 inhibited the growth of antibiotic-sensitive and -resistant *S. aureus* strains if compared to the control cultures after 10 h, whereas the single use of *Lb. rhamnosus* led to an inhibition of the germ density first after 20 h in comparison to the corresponding values of the control culture without phages. Thus, the authors suggested that phage lytic activity was predominant after 10 h of incubation and the antimicrobial activity of LAB after 20 h. The authors described the highest reduction ability for a combined application of phage and LAB after 10 h and 20 h [61]. 

In our study, the use of the PM resulted in a reduction ability already after 30 min (*p* < 0.05). It has to be pointed out that the reduction was shown at each sampling time compared to the initial values as well as to the control culture. The combination of PM and LAB also led to a reduction after 30 min, after 12 h and after 24 h, but without significance (*p* > 0.05). From this, it can be concluded that the addition of LAB did not show an increase or synergism for an incubation period of 24 h. The sole use of the LAB in the present study led to a reduction only compared to the values of the control culture without phages first after 24 h of incubation (*p* > 0.05), which is comparable to the results of Woo and Ahn (2014) and will be related to the fact that the proliferation, the synthesis of antimicrobial peptides and consequently the antimicrobial activity of LAB requires more time than that of obligately lytic phages. This could also be a possible reason explaining why the combination with LAB and the use of LAB alone did not lead to a significant result (*p* > 0.05). As with all microorganisms, there is a lag phase, where bacteria adapt to growth conditions, and so the effect of the LAB probably started when the present measurements were finished after 24 h. Therefore, an extension of the observation time would be necessary. A further in vivo trial could be used to investigate whether the LAB, by being established in the udder, could be more effective and whether a prolonged incubation period in vivo may potentially lead to a significant effect of the LAB against *S. aureus*. In any case, our results show that there are no negative interferences between the PM and the LAB which would have led to a lower reduction in the combined application, underlining the possibility of a combined use in further trials. In this combination, lytic phages could thus achieve a short-term effect, whereas the LAB could offer the advantage of a long-term effect by establishing itself in the target tissue. At this point, further examinations are necessary to investigate this context more detailed in vivo. 

Further, it is possible that incubation under anaerobic conditions would have had an influence on the antimicrobial activity of the *Lb. plantarum* strain. However, the present in vitro investigations were conducted against the background of in vivo use in the treatment of mastitis cases and as LAB represent anaerobic but aerotolerant microorganisms, the approaches were incubated under aerobic conditions.

Although the resistance situation of antibiotics for mastitis therapy in Germany can still be described as moderate, the isolation of resistant *S. aureus* strains from mastitis cases and tank milk has been published [9,62,63]. Since the development of antibiotic resistance follows a natural process and any application and in particular the improper administration of antibacterial agents can lead to the development of resistance in bacteria, alternative solutions for the treatment of bacterial infections are needed [3,5]. The use of probiotic lactic acid bacteria as well as phages in therapy represents such alternative approaches and offers several advantages. These include, among others, the protection of the apathogenic flora when using phages [21], the modulation of the immune system by LAB as a probiotic property [34] as well as a self-limitation of phages at low *S. aureus* concentrations [17] and, in contrast, a possible long-term effect of LAB by establishing themselves in the udder. There are already investigations on a combined use of phages and probiotics against different pathogens [61,64,65]. Dini et al. (2016) demonstrated pathogen reduction in the treatment of in vitro EHEC infection using the phage CA933P and a probiotic mixture, with a diminishing cytotoxic effect. The authors additionally demonstrated freeze drying as a method of storage with no significant reduction in concentration of the two components [66]. We determined a storability of the phages used in the present study for a period of six month at +6 °C without a substantial loss of efficacy (< 1 log unit), determined for pfu/mL in periodically performed plaque assays of the phage solution after propagation and sterile filtration. This storage stability represents an important factor with regard to its therapeutic use [67]. Another study investigated the use of phage P22 and a mixture of four bacteriocin-producing *Lactobacilli* for the treatment of *Salmonella* infection in chicken, resulting in the total elimination of intestinal *Salmonella* after 48 h [64]. Accordingly, the investigation of phages and LAB against pathogens is not new but, to our knowledge, the present study is the first examination of a combined use with regard to bovine mastitis therapy.

Further studies are needed to investigate the efficacy of a combined application of phages and LAB for the treatment of *S. aureus* mastitis more detailed in vivo. For this case, the combination of the phage mixture and the LAB 118/37 could be applied into the teat canal of healthy udders for a tolerability assessment, followed by the investigation of the BC rates of an intramammary treatment in comparison to treatment with common antibiotic agents. Further examinations could also deal with a prophylactic use in the cleaning of milking equipment. D’Accolti et al. (2018) already demonstrated that a combination of phages (Staphylococcal phage and Pyophage) and the probiotic PCHS detergent including spores of three Bacillus species led to decontamination of hard surfaces in the hospital environment (hospital-acquired pathogens, e.g., antibiotic-sensitive and -resistant *S. aureus*) [65]. The authors also noted that the probiotic detergent not only had a positive effect on the maintenance of phage efficacy, but also led to an increased activity compared to the suspension in PBS. Thus, they suggested the phage–probiotic combination for use as a routine disinfectant [65]. In the present study, the LAB had no adverse effect on the activity of the phages when used in combination and an extended incubation period in further investigations could possibly lead to a significant influence on the *S. aureus* germ density.

It is a fact that any use of antibiotics promotes the development of bacterial resistance and some bacterial infections, such as *S. aureus* infections, whether in animals or humans, may not respond adequately to antibiotics [5]. These problems make it necessary to research alternatives for the therapy of bacterial infections. Against the background of a limited number of studies on a combination of phages and LAB and no prior investigation into mastitis therapy, the present study provides a basis for future investigations. Nevertheless, further in vivo studies are necessary to find adequate application doses and intervals as in vitro investigations are not fully able to reproduce in vivo conditions due to the complex anatomical and physiological conditions of the bovine udder. 

## 5. Conclusions

The antimicrobial activity of a single application as well as the combination of a *Lb. plantarum* strain with proven antimicrobial properties and a lytic three-component phage mixture was investigated in milk against *S. aureus* isolates from bovine mastitis. The single application of the phage mixture showed the most efficient reduction of *S. aureus* germ density after 12 h (*p* < 0.05). The combination of the phage mixture and the lactic acid bacterium showed the highest antimicrobial activity after 24 h (*p* > 0.05). Statistical calculations showed that only the phage mixture had a significant effect on the *S. aureus* germ density for an incubation period of 24 h.

## Figures and Tables

**Table 1:** Bacteriophages and *Staphylococcus aureus* propagation strains.

Phage	Family; Relationship	Phage Origin	Propagation Strain (*S. aureus*)	Strain Origin
STA1.ST29	Myovirus; related to phage K	sewage water	ST29	human isolate
EB1.ST11	Podovirus; related to phage PSa3	pig manure	ST11	mastitis milk sample
EB1.ST27	Podovirus; related to phage PSa3	pig manure	ST27	mastitis milk sample

All phages were isolated and sequenced by PTC GmbH, Bönen, Germany, and are available upon request.

**Table 2:** Reduction of *S. aureus* germ density in log_10_ units.

Time *	PM		PM + LAB		LAB	
	A	B	A	B	A	B
30 min	1.9	1.9	2.0	2.0	0	0
12 h	5.1	8.5	3.9	7.3	+ 3.3	0.1
24 h	4.2	5.8	5.1	6.7	+ 0.1	1.5

A: compared to the initial value (5.1 log cfu/mL). B: compared to the control without phages and LAB at the same time. * Time of incubation at 37 °C in ultra-high temperature (UHT) milk. PM = phage mixture (STA1.ST29; EB1.ST11; EB1.ST27) (*p* < 0.05); LAB = lactic acid bacterium (*Lb. plantarum* 118/37) (*p* > 0.05); PM+LAB = combined application of the phage mixture and lactic acid bacterium (*p* > 0.05); + = increase in germ density. The common mean values for the reduction of the two *S. aureus* isolates 7142 and 10614 are given as the statistical calculation showed no significant difference between the two *S. aureus* isolates (*p* > 0.05).
